# Re-identification of individuals in genomic datasets using public face images

**DOI:** 10.1126/sciadv.abg3296

**Published:** 2021-11-17

**Authors:** Rajagopal Venkatesaramani, Bradley A. Malin, Yevgeniy Vorobeychik

**Affiliations:** 1 Department of Computer Science and Engineering, Washington University in St. Louis, 1 Brookings Dr., St. Louis, MO 63108, USA.; 2 Department of Biomedical Informatics, Vanderbilt University Medical Center, Suite 1475, 2525 West End Avenue, Nashville, TN 37203, USA.; 3 Department of Biostatistics, Vanderbilt University Medical Center, Suite 1475, 2525 West End Avenue, Nashville, TN 37203, USA.; 4 Department of Electrical Engineering and Computer Science, Vanderbilt University, 2201 West End Ave, Nashville, TN 37235, USA.

## Abstract

Recent studies suggest that genomic data can be matched to images of human faces, raising the concern that genomic data can be re-identified with relative ease. However, such investigations assume access to well-curated images, which are rarely available in practice and challenging to derive from photos not generated in a controlled laboratory setting. In this study, we reconsider re-identification risk and find that, for most individuals, the actual risk posed by linkage attacks to typical face images is substantially smaller than claimed in prior investigations. Moreover, we show that only a small amount of well-calibrated noise, imperceptible to humans, can be added to images to markedly reduce such risk. The results of this investigation create an opportunity to create image filters that enable individuals to have better control over re-identification risk based on linkage.

## Teaser

Face re-identification from genomic data is harder than you think!

## INTRODUCTION

Direct-to-consumer DNA testing has made it possible for people to gain information about their ancestry, traits, and susceptibility to various health conditions and diseases. The simplicity of testing services by companies such as 23andMe, AncestryDNA, and FamilyTreeDNA has drawn a consumer base of tens of millions of individuals. These sequenced genomes are of great use to the medical research community, providing more data for genome-phenome association studies, aiding in early disease diagnoses, and personalized medicine.

While genome sequencing data gathered in medical settings are anonymized and their use is often restricted, individuals may also choose to share their sequenced genomes in the public domain via services such as OpenSNP (*1*) and Personal Genome Project (*2*). Moreover, even the sharing of de-identified data for medical research typically faces tension between open sharing within the research community and exposure to privacy risks. These risks generally stem from the ability of some data recipients to link the genomic data to the identities of the corresponding individuals. One particularly acute concern raised in recent literature is in the ability to link a genome to the photograph of an individual’s face (*3*–*6*). Specifically, these studies have shown that one can effectively match high-quality three-dimensional (3D) face maps of individuals with their associated low-noise sequencing data, leveraging known associations between phenotypes, such as eye color, and genotypes, which, for the purposes of this study, correspond to the variations in our genes that affect physical traits. However, for a number of reasons, it is unclear whether these demonstrations translate into practical privacy concerns. First, the studies to date have relied on high-quality, often proprietary, data that are not publicly available. This is a concern because such high-quality data are quite difficult to obtain in practice. While many people post images of their face in public, these are generally 2D, with quality that varies considerably depending on a variety of factors, such as resolution, lighting conditions, camera angle, and background objects. Phenotype association studies, in contrast, typically use high-resolution 3D face maps captured by dedicated hardware (*3*–*5*) or photographs captured in laboratory-controlled lighting conditions to ensure minimal impact on visible features such as eye color (*6*). From a computer vision perspective, ideal datasets would have subjects directly facing the camera, with a plain background (*7*); however, this is rarely the case with images in the wild. In addition, observed phenotypes in real photographs need not match actual phenotypes, thereby making it challenging to correctly infer one’s genotype and vice versa. For example, people may color their hair or eyes (through contact lenses). Last, increasing population size poses a considerable challenge to the performance of genome-photograph linkage: Given a target individual and a fixed collection of features (the predicted phenotypes in our case), the chances of encountering others who are similar to the target individual in this feature space increase with population size. Another related study by Humbert *et al.* (*8*) investigates the re-identification risk of OpenSNP data but assumes complete knowledge of a collection of phenotypes, including many that are not observable from photographs, such as asthma and lactose intolerance. We consider this approach to be a theoretical upper bound in our study, that is, matching performance when ground-truth phenotypes are known a priori, as opposed to when predicted from face images.

Given these potential confounders in the real world, we study the risk of re-identification of shared genomic data when it can potentially be linked to publicly posted face images. To this end, we use the OpenSNP (*1*) database, along with a newly curated dataset of face images collected from an online setting and paired with a select subset of 126 genomes. We develop a re-identification method that integrates deep neural networks for face-to-phenotype prediction (e.g., eye color) with probabilistic information about the relationship between these phenotypes and single-nucleotide polymorphisms (SNPs), which are nucleotide variations distributed across the genome, to score potential image-genome matches. The first purpose of our study is to assess how substantial the average risk is, as a function of population size, given the nature of available data as well as current technology. Our second purpose is to introduce a practical tool to manage individual risk that enables those who post face images online as well as social media platforms that manage these data, to trade off risk and utility from posted images according to their preferences. We emphasize that, in the threat model invoked in this research, we consider only face images posted by individual users on social media. In this respect, the utility is implicit, as it is natural to assume that, when a user uploads a photograph to a website, they would prefer to retain as much of the original detail as possible, and visible distortions would be unwelcome in such a setting. We find that the overall effectiveness of re-identification and, thus, privacy risk is substantially lower than suggested by the current literature that relies upon high-quality SNPs and 3D face map data. While some of this discrepancy can be attributed to the difficulty of inferring certain phenotypes—eye color, in particular—from images, we also observe that the risk is relatively low, especially in larger populations, even when we know the true phenotypes that can be observed from commonly posted face images. Even using synthetically generated data that make optimistic assumptions about the nature of SNP-to-phenotype relationships, we find that the average re-identification success rate is relatively low.

Our second contribution is a method for adding small perturbations to face images before posting them, which aims to minimize the likelihood of the correct match (that is, to minimize individual risk). This framework is tunable in the sense that the user can specify the amount of noise they can tolerate, with greater noise added having greater deleterious effect on re-identification success. We show that, even using imperceptible noise, we can often successfully reduce privacy risk, even if we specifically train deep neural networks to be robust to such noise. Furthermore, adding noise that is mildly perceptible further reduces the success rate of re-identification to be no better than random guessing. We note that our privacy model here differs from common conventional models, such as *k*-anonymity (*9*–*13*) and *l*-diversity (*14*). Rather, our privacy assessment is closely tied to our risk analysis framework that combines phenotype inference from face images using machine learning with the particular approach to quantifying re-identification risk that we describe.

## RESULTS

We investigate the risk of re-identification in genomic datasets “in the wild” based on linkage with publicly posted photos. Using the public OpenSNP dataset, we identified 126 individual genotypes for which we were able to successfully find publicly posted photographs (e.g., some were posted along with genomic data on OpenSNP itself). We used a holistic approach to associate genomes to images as follows. If a user’s picture was posted on OpenSNP, higher-quality pictures could often be found under the same username on a different website. When no picture was posted for a certain user on OpenSNP, we found pictures posted on different websites under the same username and used self-reported phenotypes on OpenSNP to ensure with a reasonable degree of certainty that the image corresponds to the genome. This resulted in a dataset of SNPs with the corresponding photos of individuals, which we refer to as the Real dataset. To characterize the error rate in phenotype prediction from images, we constructed two synthetic datasets, leveraging a subset of the CelebA face image dataset (*15*) and OpenSNP. In this study, synthetic data refer to image-genome pairs that are generated by combining these two unrelated datasets, where the genome in a given pair does not correspond to the individual in the image (taken from CelebA) but comes instead from an individual with the same set of phenotypes (taken from OpenSNP). We created artificial genotypes for each image (here, genotype refers only to the small subset of SNPs we are interested in; see table S1 for the full list) using all available data from OpenSNP, where self-reported phenotypes are present. First, we consider an idealized setting where, for each individual, we select a genotype from the OpenSNP dataset that corresponds to an individual with the same phenotypes, such that the probability of the selected phenotypes is maximized, given the genotype. In other words, we pick the genotype from the OpenSNP data that is most representative of an individual with a given set of phenotypes. We refer to this dataset as Synthetic-Ideal. Second, we consider a more realistic scenario where, for each individual, we select a genotype from the OpenSNP dataset that also corresponds to an individual with the same phenotypes but, this time, at random according to the empirical distribution of phenotypes for particular SNPs in our data. Because CelebA does not have labels for all considered phenotypes, 1000 images from this dataset were manually labeled by one of the authors. After cleaning and removing ambiguous cases, the resulting dataset consisted of 456 records. We refer to this dataset as Synthetic-Realistic.

Our re-identification method works as follows. First, we learn deep neural network models to predict visible phenotypes from face images, leveraging the CelebA public face image dataset, in the form of (i) sex, (ii) hair color, (iii) eye color, and (iv) skin color. We learn a model separately for each phenotype by fine-tuning the VGGFace architecture for face classification (*16*). The result of each such model is a predicted probability distribution over phenotypes for an input face image. Second, for each input face image **x_i_** and for each phenotype *p*, we use the associated deep neural network to predict the phenotype *z*_*i*,*p*_, which is the most likely phenotype in the predicted distribution. Third, for each image **x_i_** and genotype **y_j_** corresponding to individual *j*, we assign a log-likelihood score to each image-genotype pair (**x_i_**, **y_j_**) as follows

$$p_{\mathrm{ij}}=\sum_{z_{i,p}}^{p\in \{\text{sex},\text{hair},\text{skin},\text{eye}\}}\text{log}P(z_{i,p}|y_{j})$$ (1)

This approach is similar to the one introduced by Humbert *et al.* (*8*) but differs in that we predict phenotypes from face images as opposed to assuming complete knowledge. Last, armed with the predicted log-likelihood scores *p_ij_* for genotype-image pairs, we select the top *k*-scored genotypes for each face image, where *k* is a tunable parameter that allows a precision-recall trade-off in the matching predictions.

The effectiveness of re-identification is strongly related to both the choice of *k* above, as well as the size of the population that one is trying to match against. More specifically, as we increase *k*, one would naturally expect recall (and, thus, the number of successful re-identifications) to increase. On the other hand, a larger population raises the difficulty of the task by increasing the likelihood of spurious matches. We therefore evaluate the impact of both of these factors empirically.

### Average re-identification risk is low in practice

We evaluate the effectiveness of re-identification attacks using two complementary measures: (i) the fraction of successful matches and (ii) the area under the receiver operating characteristic (ROC) curve (AUC). The former enables us to study re-identification success (while focusing on recall) as a function of population size, while the latter paints a more complete picture of the trade-off between true-positive and false-positive rates.

First, we consider the proportion of successful matches as a function of population size, i.e., the number of individuals in the genomic database. To do this, we consider several fixed values of *k*, where a match from a face image **x_i_** to a genome **y_j_** is considered successful if the associated log-likelihood score *p_ij_* is among the top *k* for the image **x_i_**.

**Fig. 1.**
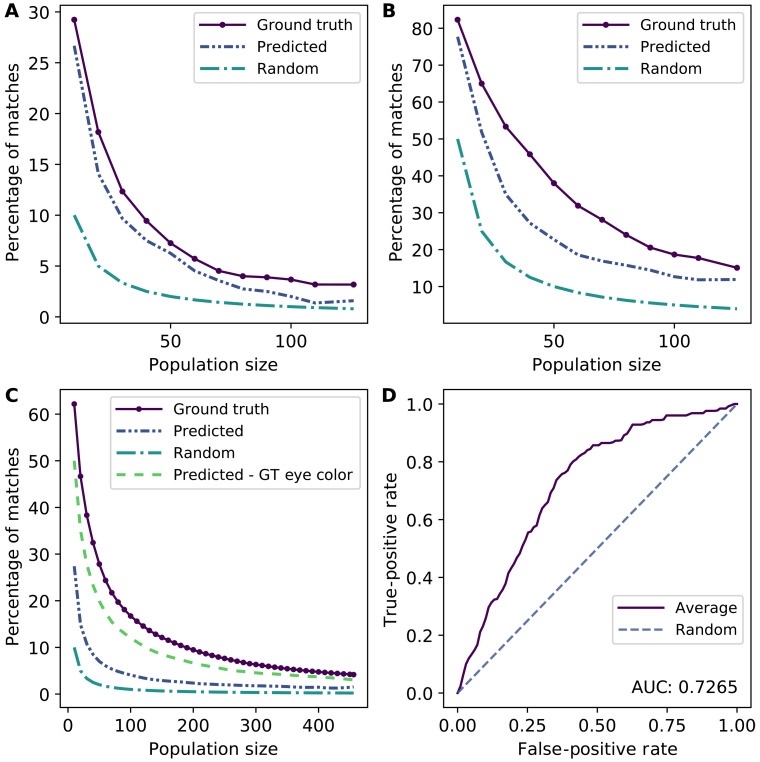
Effectiveness of matching individuals’ photos to their DNA sequences in OpenSNP. (**A**) Success rate for top 1 matching for the Real dataset. (**B**) Success rate for top 5 matching for the Real dataset. (**C**) Success rate for top 1 matching in the Synthetic-Ideal dataset. (**D**) ROC curve for 126 individuals. (A) to (C) present matching success results as a function of the population size (the number of individual genomes to match a face image to) for a fixed *k*.

The results for the Real dataset for *k* = 1 and *k* = 5 are shown in Fig. 1 (A and B, respectively). We compare the success of our re-identification approach to two baselines: (i) when matches are made randomly (a lower bound) and (ii) when matches use actual, rather than predicted, phenotypes (an upper bound). We can see from Fig. 1A that matching success (where we solely take the top-scoring match) is relatively low even for the upper bound, where we actually know the phenotypes (and, consequently, do not need the images). Nevertheless, the top 1 matching success rate is close to the upper bound (which assumes perfect knowledge of phenotypes) and is considerably better than random. As expected (*3*), prediction efficacy declines as population size grows. Figure 1C shows that, in an idealized setting, re-identification accuracy can be considerably higher; however, effectively predicting eye color is crucial, and this appears to be a major limitation of existing techniques. Figure 1D shows that, when we treat matching as a binary prediction problem, the effectiveness is well above that achieved by randomly guessing. Nevertheless, re-identification risk in the wild does not appear to be especially high. While we observe a success rate as high as 25%, this is only achieved when the genomic dataset is extremely small, on the order of 10 individuals. In contrast, success rate for top 1 matching drops quickly and is negligible for populations of more than 100 individuals. Moreover, it should be kept in mind that this result assumes that we can predict the phenotypes perfectly.

The overall pattern does not substantially change when *k* = 5. However, in this case, the matching success rates naturally increase, approaching 80% for small populations and slightly below 20% for populations of more than 100 individuals. In this case, we do observe that our re-identification approach, while significantly better than random, is also considerably below the theoretical upper bound. This suggests that, when more than a single re-identification claim is permitted for each image, the error in phenotype prediction from face images has a greater influence.

Next, we delve more deeply into the nature of re-identification risk using the larger synthetic datasets. We present the results for Synthetic-Ideal in Fig. 1C. Additional results for both the Synthetic-Ideal and Synthetic-Real datasets when the top 1,3, and 5 matches are predicted to be true matches are provided in fig. S7 (A to F). These results offer two insights. First, if an attacker has access to particularly high-quality data, re-identification risk can be relatively high for small populations. For example, the upper bound is now more than 60% in some cases. However, it can also be seen that, of the phenotypes we aim to predict, eye color is both the most difficult and highly influential in matching. If we assume that we know this phenotype, and we only have to predict the others, re-identification risk is near its upper bound (which assumes that we know the true phenotypes). This is even more notable in the case of the Synthetic-Real data, as shown in fig. S10 (A to D). To determine whether this result was an artifact of the specific method we selected for eye color prediction, we considered several alternative methods for eye color prediction (*17*), ranging from traditional computer vision techniques to deep learning [see fig. S10 (E to G)]. None of these methods were particularly efficacious.

Next, we turn our attention to a different manner of evaluating prediction efficacy: the trade-off between false-positive and false-negative rates obtained as we vary *k*. The results, shown in Fig. 1D for a population size of 126 individuals, suggest that the overall re-identification method is relatively effective (AUC > 70%) when viewed as a binary predictor (match versus non match) for given genome-image pairs, particularly when compared to random matching. ROC curves when thresholding on *k* for various population sizes are presented in fig. S1 (A to L), while, in fig. S1 (M to X), we also consider a common alternative where we use a tunable threshold θ on the predicted log-likelihood to claim when a match occurs.

Overall, our results suggest that it is sometimes possible to link public face images and public genomic data, but the success rates are well below what prior literature appears to suggest, even in idealized settings. We believe that there are several contributing factors behind this observation. First, the quality of face images in the wild is much lower than the high-definition 3D images obtained in highly controlled settings in prior studies (*3*–*6*). Second, there is a relative scarcity of high-quality training and validation data for this particular task. While there are large, well-labeled datasets for face classification (*16*, *18*–*22*), the data needed for re-identification require paired instances of genomes and images, which are far more challenging to obtain at scale. Third, visible phenotypes are influenced by factors other than just the SNPs that are known to have a relationship with them, particularly when you add artificial factors, such as dyeing one’s hair or wearing tinted contact lenses, which introduce considerable noise in the matching. Last, our analysis assumed (as did all prior studies) that we already know that there is a match in the genomic dataset corresponding to each face. In reality, success rates would be even lower, because a malicious actor is unlikely to be certain about this (*23*).

### Achieving privacy through small image perturbations

While the assessment above suggests that the re-identification risk to an average individual is likely lower than what has been suggested in the literature, it is nevertheless evident that some individuals are at risk. Moreover, if the attacker has sufficient prior knowledge to narrow down the size of the population to which a face can be matched, our results do show that, even on average, the re-identification risk becomes non-negligible. This led us to investigate the natural question: How can we most effectively mitigate re-identification risks associated with the joint public release of both genomic data and face images? Our specific goal is to provide tools that can reduce re-identification risks to individuals who publicly post their photos. Such tools can be used directly either by individuals to manage their own risks or by platforms where photos are posted to manage risks to their subscribers. In particular, we show that this can be accomplished by adding small image perturbations to reduce the effectiveness of genomic linkage attacks.

**Fig. 2.**
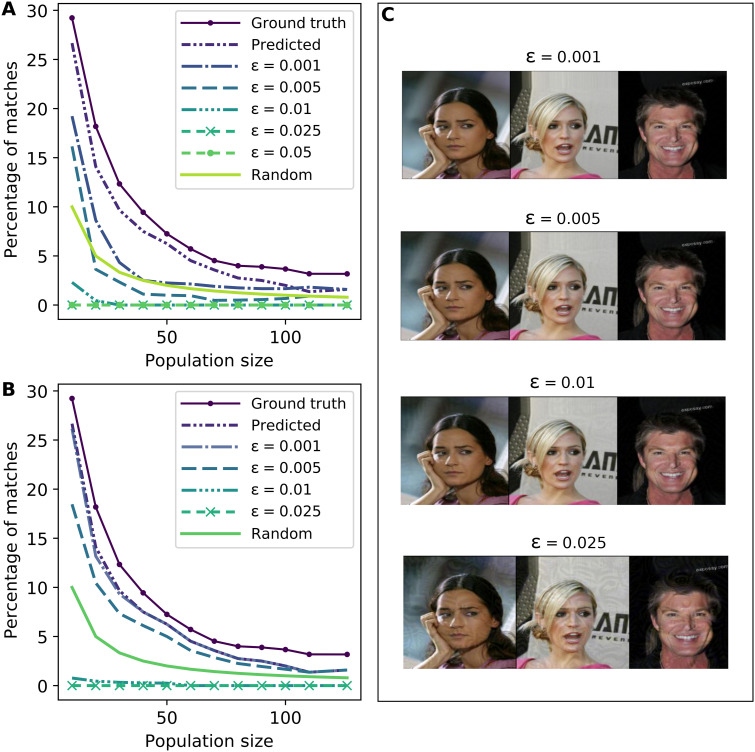
Evaluating small image perturbations as a defense. (**A**) Effectiveness of perturbations as a defense against re-identification for *k* = 1 (i.e., the attacker considers only the top match). Pixel values are normalized to a [0,1] interval, and perturbation strengths ϵ are with respect to these normalized pixel values. It can be seen that prediction accuracy is near zero at a perturbation strength ϵ ≥ 0.01. Moreover, even for very small amounts of adversarial noise, such as ϵ = 0.001, matching success is nearly indistinguishable from random matching if we have at least 20 individuals in a consideration pool. (**B**) Effectiveness of perturbations that only target sex prediction from a face image. The effect of larger perturbations (ϵ ≥ 0.01) is similar to (A). However, smaller perturbations are considerably less effective. (**C**) Example images [photo credit: The CelebA Face Dataset (*15*)] illustrate the visible effect of introducing small perturbations to images. The perturbations are essentially imperceptible to a human until ϵ > 0.01, when the effect becomes clearly visible.

Our approach is closely related to adversarial perturbations in computer vision (*24*, *26*). The idea behind adversarial perturbations is to inject a small amount of noise into an image to cause a misprediction, where “small” is quantified using an *l_p_* norm with the maximum allowable perturbation controlled by a parameter ϵ ranging from 0 to 1. Examples of the visual impact of increasing ϵ are shown in Fig. 2C and fig. S3. In our case, however, we do not have a single deep neural network making predictions, but rather a collection of independent phenotype predictors using the same image as input. One direct application of adversarial perturbations in our setting would be to arbitrarily choose a phenotype (say, sex, which is the most informative) and target this phenotype for misprediction, with the anticipation that this would cause re-identification to fail. However, we can actually do much better at protecting privacy by tailoring perturbations to our specific task. This is because our ultimate goal is not to cause mispredictions of phenotypes per se but, rather, to cause an attacker to fail to link the image with the correct genome.

Specifically, we leverage the scoring function in Eq. 1 to minimize the score *p_ij_* for image **x_i_** and the correct corresponding genome **y_j_**. However, this is a nontrivial task because the scoring function has a discontinuous dependence on predicted phenotypes (because we use only the most likely phenotype, given an image, in computing it). To address this issue, we augment the score with the log of the predicted probabilities. More precisely, let *g_p_*(*v_p_*, **x_i_**) denote the probability that the neural network predicts a variant *v_p_* for a phenotype *p* (e.g., eye color) given the input face image **x_i_**. Our objective is to find a small perturbation to the input image δ^*^ that solves the following problem

$$\underset{-\epsilon \leq \delta \leq \epsilon }{\text{min}}\sum_{p}^{p\in \{\text{sex},\text{hair},\text{skin},\text{eye}\}}\sum_{v_{p}}\text{log}\;g_{p}(v_{p},x_{i}+\delta )\text{log}P(v_{p}|y_{j})$$ (2)

where ϵ refers to the maximum allowed perturbation to each pixel, as is common in prior approaches for finding adversarial perturbations (*24*, *25*). Because this expression is differentiable with respect to the adversarial noise δ, we can solve it using standard gradient-based methods (see Materials and Methods for further details).

Our first evaluation, shown in Fig. 2, presents the effectiveness of our method for preserving privacy in public face images. Figure 2A, in particular, demonstrates that when we take deep neural networks for phenotype prediction as given, the effectiveness of the re-identification attack described above declines considerably even for very small levels of noise added to images. For sufficiently large noise (e.g., ϵ = 0.01), the success rate is close to zero, which is considerably lower than random matching. Moreover, by comparing Fig. 2A to Fig. 2B, it can be seen that our approach is also more effective than designing small perturbations that target a single sex phenotype. The effectiveness of targeting other phenotypes is provided in fig. S2 (D to F), where it can be seen that perturbations that target only hair color, eye color, or skin color predictions are insufficient to induce a substantial level of re-identification risk reduction. While the presented results are only for *k* = 1 (i.e., the attacker only considers the top-scoring match), results for *k* = 3 and *k* = 5 offer similar qualitative insights (as shown in fig. S4).

The visual effect of the designed image perturbations is illustrated in Fig. 2C using images drawn from the public celebrity face image dataset. As can be seen, most of the levels of added noise have negligible visual impact. It is only when we add noise at ϵ = 0.025 that we begin to clearly discern the perturbations. However, it appears that perturbations of magnitude no greater than ϵ = 0.01 are sufficient to achieve a high degree of privacy, with success rates of re-identification attacks nearing zero.

While introducing small perturbations appears to sufficiently reduce the risk of re-identification introduced by publicly shared photographs, this still supposes that re-identification makes use of deep neural network models trained in the regular fashion, such as on the CelebA dataset. However, a variety of techniques allow one to boost neural network robustness to such perturbations, the most effective of which is adversarial training (*25*, *26*). The adversarial training approach improves robustness by augmenting each iteration with training images that actually embed the small perturbations we have designed, using the model from the previous iteration as a reference for devising the perturbations. The main limitation of adversarial training is that it also results in lower accuracy on data that have not been perturbed. Given the relatively limited effectiveness of the re-identification approach above and all of the practical limitations of that exercise, we now investigate whether, in practice, adversarial training helps the attacker deflect the small perturbations we introduce to protect privacy.

To evaluate the effect of adversarial training on re-identification, we run further training iterations on the phenotype-prediction models with small perturbations generated over subsets of the original training sets. We make five passes over the perturbed data, each time using the model from the previous iteration to generate these small perturbations to the images. Because the matching score depends on images and corresponding genomes, we use paired genome-image datasets for adversarial training (the most optimistic setting from the re-identification perspective). Specifically, we use a random subset of 77 image-DNA pairs (approximately 60%) from our OpenSNP dataset for training, and the remaining 49 for testing the matching accuracy. We construct five sets of adversarially robust phenotype prediction models using this procedure, each set adversarially trained using a different amount of added adversarial noise, from ϵ = 0.001 to ϵ = 0.05.

**Fig. 3.**
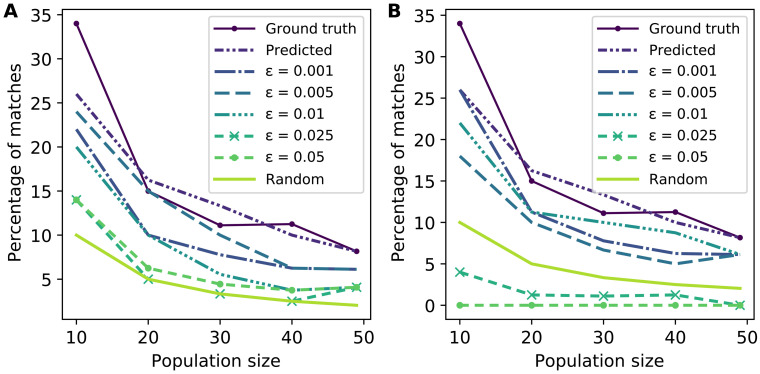
Evaluation of models that are trained to increase robustness to small perturbations through adversarial training when only the top match is considered in re-identification. (**A**) Matching accuracy of “robust” models trained by adding perturbations with varying levels of ϵ when unperturbed face images are used as inputs. Using ϵ > 0.01 causes matching accuracy to be effectively equivalent to random. (**B**) Matching accuracy of robust models trained by adding perturbations with ϵ = 0.01 when input face images are perturbed with varying levels of adversarial noise. Using ϵ > 0.01 is sufficient to cause sub-random matching accuracy. For noise with ϵ = 0.01, matching accuracy degrades from original but remains higher than random.

Figure 3A illustrates that baseline prediction accuracy (i.e., using original face images without perturbations) declines as strength of the perturbation used for adversarial training increases. Once ϵ > 0.01, the effectiveness of matching is essentially equivalent to random, suggesting that the most robust model that holds any utility is the one with ϵ = 0.01.

Next, we consider how robust this model is to images that now include small perturbations of varying magnitudes generated using the procedure we describe above. The results are shown in Fig. 3B. Notably, noise with ϵ = 0.025 again yields near-zero matching success, even for robust models. A smaller amount of noise that preserves imperceptibility, such as ϵ = 0.01, is still effective at reducing the accuracy of the robust model, although the resulting re-identification success rate is now above random matching. Nevertheless, re-identification success in this case is ∼20% even in the most optimistic case. Moreover, our framework is sufficiently flexible that a particularly risk-averse individual may simply raise the noise level to 0.025, accepting some visible corruption to the image but effectively eliminating privacy risk.

## DISCUSSION

Our findings suggest that the concerns about privacy risks to shared genomic data stemming from the attacks matching genomes to publicly published face photographs are low and relatively easy to manage to allay even the diverse privacy concerns of individuals. Our results do not imply that shared genomic data are free of concern. There are certainly other potential privacy risks, such as membership attacks on genomic summary statistics (*27*–*35*), which would allow the recipient of the data to determine the presence of an individual in the underlying dataset. This type of attack is of concern because it would allow an attacker to associate the targeted individual with the semantics inherent in the dataset. For instance, if the dataset was solely composed of individuals diagnosed with a sexually transmitted disease, then membership detection would permit the attacker to learn that the target had the disease in question. Moreover, we emphasize that our results are based on current technology; it is possible that improvements in either the quality of data, such as broad availability of high-definition 3D photography, or the quality of artificial intelligence, such as highly effective approaches for inferring eye color from images, will substantially elevate risks of re-identification. In recent literature, approaches to certify robustness against adversarial examples using various mechanisms, such as randomized smoothing (*36*–*38*), have been leveraged to further boost the robustness of neural networks to adversarial perturbations and could further contribute to increased risks of re-identification. However, through several studies that include synthetic variants controlling for the quality of data, as well as evaluations that assume that we can infer observable face phenotypes with perfect accuracy (see, for example, the results in Fig. 1A and in the figs. S7 and S10), we show that, even with advances in technology, the risk is likely to remain limited.

## MATERIALS AND METHODS

### Re-identification attack with public face images

In our attack, we consider the following phenotypes to be readily visible in face images: eye color, hair color, sex, and skin color. To this end, we first collected genomes uploaded to OpenSNP that were sequenced by 23andMe. We then filtered the users who have self-reported all four phenotypes we are interested in. Some of these users also uploaded their face images directly on OpenSNP. For others, we found their faces through a Google reverse search using their OpenSNP usernames, which we then manually verified using the self-reported phenotypes. This process yielded 126 pairs of face images and DNA profiles of individuals that were carefully curated. The full study was approved by the Washington University in St. Louis Institutional Review Board, with the condition that we will not publicly release this curated dataset to mitigate any possible harm to these individuals. However, we are able to share it privately with other researchers for validation purposes, subject to an agreement preventing public release.

### Predicting phenotypes from images

To predict phenotypes from facial images, we leveraged the VGGFace (*16*) convolutional neural network architecture. Because of the relative scarcity of labeled training data for phenotypes of interest, we used transfer learning (*39*), which has been extensively and successfully used for various classification tasks where labeled data are scarce (*40*–*47*). Specifically, we started with a model previously trained on the CelebA dataset (*15*) for a face recognition task. We then fine-tuned these models on subsets of the CelebA dataset. For sex and hair color, the CelebA dataset already contains labels for all ∼200,000 images, and we thus used the entire dataset to fine-tune the sex prediction classifier. For hair color, we found that fine-tuning on a subset of 10,000 images with equal number of blonde, brown, and black hair color images outperforms a model trained on the entire dataset; we thus used the latter. For skin color, 1000 images were labeled on a five-point scale by Amazon Mechanical Turk (AMT) workers and then manually sorted into three classes. For eye color prediction, 1000 images were labeled by AMT workers; however, after manual verification of these labels, we retained ∼850 images, dropping the rest because the eye color was indeterminate.

### Matching faces to DNA

To match faces to genomic records, we use the following procedure. After learning phenotype classifiers for each visible phenotype, we predict the most likely variant (i.e., the one with the largest predicted probability) for each face image in test data. We then use this prediction for matching the face image to a DNA record as follows. Let an image be denoted by **x_i_** and a genome by **y_j_**. We use the following matching score, where phenotype variant *z*_*i*,*p*_ is the most likely predicted variant

$$P_{\mathrm{ij}}=\prod_{p}^{p\in \{\text{sex},\text{hair},\text{skin},\text{eye}\}}P(z_{i,p}|y_{j}).$$ (3)

To ensure numerical stability, we transform it into log space, resulting in

$$p_{\mathrm{ij}}=\text{log}\;P_{\mathrm{ij}}=\sum_{p}^{p\in \{\mathrm{sex},\text{hair},\text{skin},\mathrm{eye}\}}\text{log}\;P(z_{i,p}|y_{j})$$ (4)

The variant *z*_*i*,*p*_ is predicted from {F, M} for sex, {blue, brown, intermediate} for eye color, {black, blonde, brown} for hair color, and {pale, intermediate, dark} for skin color. The probability of each phenotype variant given a genome *P*(*z*_*i*,*p*_∣**y_j_**) is, in turn, expressed as a product of probabilities over relevant SNPs (see table S1 for these lists). The probability of a specific phenotypic variant given a certain SNP is calculated empirically from all OpenSNP data for which the corresponding individuals self-reported all four phenotypes considered in the study. There were 367 such individuals, including the 126 individuals from the Real dataset to ensure a sufficient amount of data.

Having calculated the likelihood of a match between image **x_i_** and DNA sequence **y_j_** for all images, for all DNA sequences, we rank the DNA sequences in decreasing order of matching likelihood for each image. The presented results correspond to when the correct match resides in the top-scored 1,3, or 5 entries in this sorted list.

### Protecting privacy by adding noise to face images

Recall that our goal is to minimize the score *p_ij_*, where **x_i_** is the image and **y_j_** is the correct corresponding genome. Because the score function has a discontinuous dependence on phenotype predictions, we augment the score with the log of the predicted phenotype probabilities. Specifically, let *g_p_*(*v_p_*, **x_i_**) denote the probability that the neural network predicts a variant *v_p_* for a phenotype *p* (e.g., eye color) given the input face image **x_i_**; these are just the outputs of the corresponding softmax layers of the neural networks. The problem we aim to solve is to find a small (at most ϵ in the *l*_∞_ norm ) perturbation to the input image δ^*^ that solves the following problem

$$\underset{-\epsilon \leq \delta \leq \epsilon }{\text{min}}\sum_{p}^{p\in \{\text{sex},\text{hair},\text{skin},\text{eye}\}}\sum_{v_{p}}\text{log}\;g_{p}(v_{p},x_{i}+\delta )\text{log}\;P(v_{p}|y_{j})$$ (5)

We use projected gradient descent to solve this problem, invoking a combination of automated differentiation in PyTorch (*48*) and the Adam optimizer (*49*). After each gradient descent step, we simply clip the noise to be in the [−ϵ, ϵ] range and also clip pixel values to ensure that these remain in a valid pixel value range. We use the original image as the starting point of this procedure (i.e., initializing δ = 0).

### Training robust phenotype classification models

While the idea of adding small noise as a privacy protection mechanism works well when we use regularly trained phenotype prediction models, one can make such models more robust, albeit at a cost to accuracy on noise-free data. As such, we investigate how effective adversarial training, a state-of-the-art approach for making predictions robust to adversarial noise, is at overcoming our noise injection approach.

The main premise of adversarial training is as follows. The broad goal is to solve the following optimization problem

$$\underset{\theta }{\text{min}}\sum_{x,y\in D}L(\theta ,x+\delta^{*}(\theta ),y)$$ (6)

where δ^*^(**θ**) is the adversarially induced noise aimed at the model with parameters **θ**, and *L*( · ) is the loss function. In practice, computing an optimal noise to add is difficult, and we instead use the approaches such as projected gradient descent described above. However, adversarial training proceeds like regular training (using gradient descent), except that each training input is **x** + δ^*^(**θ**) rather than **x**, that is, we use perturbed inputs in place of regular inputs.

A standard way of achieving empirically robust models is to simply augment training data with perturbations generated over a subset of the training data. In generating these instances, we use random starting points for generating such perturbations. The downside of adversarial training, however, is that robustness to adversarial inputs often comes at the cost of accuracy on unaltered inputs, and a careful balance must be achieved between adversarial robustness and baseline accuracy.

## Notes

View/request a protocol for this paper from *Bio-protocol*.
