# Supplementary material for: Re-identification of individuals in genomic datasets using public face images
Source: Sci Adv. 2021 Nov 17;7(47):eabg3296. doi: 10.1126/sciadv.abg3296 (PMC8597988; doi:10.1126/sciadv.abg3296)
Supplement: Supplementary file 1 — Supplementary Text Table S1 Figs. S1 to S10 [file sciadv.abg3296_sm.pdf]

Science Advances
AAAS

Supplementary Materials for

Re-identification of individuals in genomic datasets using public face images

Rajagopal Venkatesaramani*, Bradley A. Malin, Yevgeniy Vorobeychik

*Corresponding author. Email: rajagopal@wustl.edu

Published 17 November 2021, *Sci. Adv.* **7**, eabg3296 (2021)
DOI: 10.1126/sciadv.abg3296

This PDF file includes:

Supplementary Text
Table S1
Figs. S1 to S10

Introduction to the Supplement

This supplement provides additional methods, descriptions and results for our study, Re-identification of Individuals in Genomic Datasets Using Public Face Images. Section 1 outlines the SNPs and corresponding phenotypes considered in our study. Section 2 presents a brief overview of the deep neural network architecture used for phenotype extraction from face images. We present results when treating the re-identification process as a binary prediction problem - Receiver Operating Characteristic curves for various population sizes when the prediction threshold is varied - in Section 3, and Section 4 illustrates the use of a Support Vector Machine (SVM) to make binary matching predictions. While the main body focuses primarily on re-identification

risk, and steps taken to mitigate it in the top-1 case, i.e., only the genome with the maximum calculated log-likelihood is predicted to be a true match, in the supplement, we also present results in the top-3 and top-5 cases. Sections 5 and 6 contain results for adding small perturbations to images to protect privacy of individuals posting their face photographs publicly, and the effects of adversarial training. Additionally, when attacking a single-phenotype, the main body focuses on the prediction of sex from face images; results for the other phenotypes - namely skin color, hair color and eye color - as well as top-3 and top-5 results when attacking sex prediction are explored here. Finally, Section 7 elaborates upon our experiments with the two synthetic datasets referred to in the main body, with particular emphasis on the impact of eye color prediction on the matching pipeline.

1 SNP-Phenotype Associations

Single Nucleotide Polymorphisms (SNPs) are variations in an individual's DNA that are probabilistically linked to various phenotypes. In the context of our study, the phenotypes we are interested in are the individual's sex, skin color, eye color and hair color. To associate phenotypes predicted from images to genomes, we consider the same set of SNPs as used in study (*8*) to produce what we refer to as the upper-bound "ground-truth" baseline for the relevant phenotypes. The SNPs considered in the study are presented in Table S1. In computing the matching score, the empirically calculated prior probability of a phenotypic variant is used where SNPs are missing from an individual's genotype.

2 Matching accuracy with varying prediction thresholds - Receiver Operating Characteristic (ROC) curves

When predicting that an image matches with a particular genome, we can use thresholds to make predictions in two different ways. The first method is by thresholding with an integral value k, where we predict a positive match when a selected genome is in the top-k potential matches by likelihood, given an image. Alternatively, we can predict a positive match for each image-DNA pair if the matching likelihood is above a real valued threshold θ. These approaches complement each other, in the sense that in the former case, we understand the false positive vs. true positive rate tradeoff in terms of narrowing potential matches down to a likely sub-population, whereas the latter allows us to understand the performance of making independent predictions for image-DNA pairs. Fig. S1 (A)-(L) show the precision recall curves for various population sizes, when k is

increased from 1, where a single match is predicted, to the population size where everyone is predicted to be a match. Similarly, Fig. S1 (M)-(X) show ROC curves for various population sizes, when the threshold θ is increased from 0 to 1 in real-valued increments. In both cases, we observe that the classifier performs better than a random-guessing baseline, although the area under the curve remains relatively low, around 0.7 for the top-k method, and 0.6 when making independent predictions.

3 SVM - Matching as Binary Classification

For the sake of completeness, we also study the use of classical machine learning methods to predict matches between images and DNA, given a vector representing the likelihood of phenotypes detected in an image corresponding to a selected genome. We train a linear SVM with equal number of true and false matches, selected from subsets of the 126 individuals (10-fold cross-validated). From results in Fig. S2 (A)-(C), we see that this approach does not contribute much to matching accuracy, likely arising from limited signal present in the small dataset. Linear SVM was found to outperform SVM with a non-linear (rbf) kernel, as well as several other learning methods.

4 Protecting Privacy with Adversarial Noise

To defend against potential re-identification by a malicious actor, we propose adding small perturbations, akin to adversarial examples (*22*), as a means to preserve privacy. Here, adversarial noise is calculated using gradient based methods to minimize the matching log-likelihood. We present results for both directly solving this minimization using projected gradient descent (PGD) where the noise is calculated over all phenotype classifiers (*Universal Noise* attack), as well as using PGD to attack one phenotype classifier at a time by maximizing the corresponding neural network's cross entropy loss. The former was described in the main body, and the impact of increasing the maximum allowable perturbation per-pixel is shown in Fig. S3. We now briefly review the PGD attack targeting a single phenotype at a time (e.g., sex phenotype prediction); see (*21*) for further details.

Recall that $g_p(v_p, x_i)$ denotes the probability that a phenotype variant v_p is predicted from image x_i. Slightly abusing notation, let $g_p(x_i)$ be the probability distribution over variants given an input x_i. Let $L(g_p(x_i), y_p)$ be the loss associated with the true variant y_p and predicted variant distribution $g_p(x_i)$. The goal of the PGD

approach for generating adversarial noise is to maximize loss:

$$\max_{-\epsilon \leq \delta \leq \epsilon} L(g_p(x_i + \delta), y_p).$$

We can do this by using a form of gradient descent. Specifically, let δ_k be the value of noise in iteration k (starting with $\delta_0 = 0$, or a small random noise). Then

$$\delta_{k+1} = \delta_k + \alpha \ sgn \nabla \mathcal{L}(g_p(x_i + \delta_k), y_p),$$

where α is the learning rate. This process is run for a fixed number of iterations, or until convergence.

Results for top-1 matching when attacking sex are explored in the main body of the paper, while here in the supplement, we first explore the scenario when other phenotypes are attacked independently, and when the correct match lies within the top-3 or top-5 most likely genomes. Consider first, the top-1 matching accuracy when attacking eye-color, hair-color and skin-color predictions using PGD in Fig. S2 (D)-(F). While attacking with increasing values of ϵ does degrade performance compared to clean images, these attacks are not as effective as fooling the sex-prediction model. Independently, these phenotypes do not seem to contribute greatly to matching accuracy, in contrast to sex, where accuracy drops below random for small populations for $\epsilon = 0.01$. This problem is addressed in the *Universal Noise* setting where all phenotypes are attacked in parallel by directly minimizing the matching log-likelihood.

Next, we look at the top-3 and top-5 matching accuracy for attacking all phenotypes individually, as well as the *Universal Noise* attack. The random baseline in these cases makes 3 and 5 random guesses out of the population in question respectively. This approach illustrates the scenario when a malicious actor is trying to narrow down an individuals genome down to a small subset of the population as possible matches. Fig. S4 (A)-(E) present results in the top-3 case and Fig. S4 (F)-(J) present results in the top-5 case. Naturally, performance improves compared to the top-1, albeit at the cost of an increase in the number of false positives (k or $k-1$ false positives per image). Similar to the results in the top-1 case, attacking sex, and the *Universal Noise* approach prove to be highly effective, while independently attacking other phenotypes does not drop accuracy below random for any population size.

5 Adversarial Training

We next investigate if our adversarial noise defense may be impacted if the malicious actor in question trains phenotype-prediction models robust to such perturbations. A common way of doing this is adversarial training,

where training is augmented with adversarial examples. First we look at the effect of retraining on adversarially perturbed images for various attack strengths. We use a value of $\epsilon = 0.01$ to train each classifier, as our evaluation show it to be a somewhat optimal point, with regards to the tradeoff between effectiveness as a defense and perceptibility of image perturbation. Fig. S5 (A)-(D) show top-1 matching results for phenotype classifiers adversarially trained at $\epsilon = 0.01$ and attacked at various values of ϵ ranging from 0.001 to 0.05. Similarly, Fig. S6 (A)-(D) show top-3 matching results.

In case of sex and skin color, adversarial training boosts robustness to perturbed images as expected, it makes no difference, or even degrades performance slightly for eye color and hair color prediction. The slight loss in performance on adversarial examples is somewhat unusual, and most likely due to limited training data. Adversarial training against the *Universal Noise* approach boosts accuracy for small populations, but quickly falls to zero as population size approaches 50.

Next we are interested in how adversarial training affects baseline performance, i.e., matching accuracy on clean images. It is typical for baseline performance to degrade for more robust models. Fig. S5 (E)-(H) and Fig. S6 (F)-(J) present top-1 and top-3 matching results respectively for various values of ϵ at which the model was adversarially trained, but using clean images. In all cases, we observe a performance decrease, compared to the original models' performance on the same clean images. We observe that performance degrades to the point where adversarial training may be more detrimental than beneficial to a malicious actor attempting re-identification on a genomic dataset, especially in the case of sex classification, where training against sufficiently strong adversarial noise ($\epsilon = 0.025$) reduces accuracy to below random guessing. In case of the *Universal Noise* approach, while accuracy remains above random, note that retraining was only run for a very small number of data-points, due to the lack of paired image-DNA data, which is not required when individually retraining phenotype classifiers against PGD.

6 Evaluation on a Synthetic Dataset

While our results on the OpenSNP dataset portray a realistic picture of what the risk of re-identification is, given publicly available data, we wish to also test how our approach performs on a larger dataset of higher quality - essentially controlling for model transfer error in the transfer-learning process, while accounting for much larger population sizes. To accomplish this, we create a synthetic dataset using the CelebA dataset, by predicting genomes using existing OpenSNP data. We consider two settings - (A) an 'ideal' scenario where

each image is assigned a genome that maximizes the probability of the phenotypes detected in the image (*Synthetic-Ideal*), and (B) a 'realistic' scenario where genomes are randomly picked from a subset of OpenSNP individuals with the same set of phenotypes (*Synthetic-Realistic*). Note that we do not require the OpenSNP individuals to be of the same sex in this synthesis process due to data sparsity - the considered facial phenotypes being independent of sex is a reasonable assumption. As not all phenotypes are labeled in the CelebA dataset, we start with 1000 manually annotated images, and after cleaning and removing ambiguous cases, we are left with 456 individuals in the synthetic dataset.

We run similar evaluations on these two synthetic datasets as we did on the 126 OpenSNP individuals - matching accuracy (Fig. S7 (A)-(F)), attacking the sex classifier with PGD (Fig. S7 (G)-(L), we refrain from evaluating for other phenotypes as they proved to be largely ineffective on the OpenSNP data), baseline performance of an adversarially trained sex classifier (Fig. S8 (A)-(F)), robustness of the adversarially trained sex classifier to perturbed images (Fig. S8 (G)-(L)), and attacking with the *Universal Noise* (Fig. S9).

Next, we seek to explain the significantly larger gap between the accuracy of predicted matches and the accuracy with ground-truth phenotypes in case of the synthetic datasets. Experiments reveal that this behavior is rooted in the poor performance of our eye-color classification model, owing to both sparsity and low-quality of data available for training, and the fact that eye-color problem remains an open problem in computer vision - an especially hard one at that. Having evaluated multiple approaches (including segmentation of the eyes and classic machine learning methods with color histograms), we did not have much success in improving our predictions for this particular phenotype. The following are its implications to matching accuracy. We present as evidence two complementary sets of results - matching accuracy when eye color is entirely ignored, and matching accuracy when predicted variants from images are used for all phenotypes but for eye color, where we swap in favor of the ground-truth variants instead.

Results in Fig. S10 (A)-(D) confirm our suspicions. Ground truth accuracy drops significantly when eye-color is entirely disregarded, signalling the importance of the phenotype in matching, while our predicted accuracy slightly increases upon disregarding eye-color, signalling high volume of noise in our predictions. Subsequently, replacing our eye-color predictions with ground truth values produce accuracies that are nearly the ground-truth upper bound, strongly indicating our eye-color prediction models act as a bottleneck in the matching pipeline.

We consider a number of techniques to address this issue, ranging from conventional machine learning to neural networks. Because eyes are a small fraction of a face image, it could be very likely that the rest of

the image makes eye color prediction harder. To understand the impact of this, we segment the eyes from each image using Multitask Cascade CNNs (*13*), and use each approach on the segmented eyes (with the exception of VGGFace, which was designed specifically to work on full face images). From conventional machine learning, we report results using k-Nearest-Neighbors and a Support Vector Machine (SVM) with the rbf kernel, which outperform others with test accuracies of 57.89% and 51.75% respectively. We also build a shallow convolutional neural network, whose accuracy seems to be high at 60%. Unfortunately, this CNN converges to always predicting the majority class (Brown eyes), leading to its seemingly high performance. By contrast, the VGGFace classifier achieves a test accuracy of 59%.

Fig. S10 (E)-(G) show re-identification performance when using each approach to predict eye color. None of the approaches are particularly different from the VGGFace classifier. Future advances in computer vision could solve this problem, proposing an increased risk of re-identification in the wild.

**Phenotype**	**Traits**	**SNPs**
Sex	M<br>F	Sex Chromosome
Skin Color	Pale<br>Int<br>Dark	rs26722<br>rs1667394<br>rs16891982
Hair Color	Blonde<br>Brown<br>Black	rs12821256<br>rs35264875
Eye Color	Blue<br>Brown<br>Int	rs916977<br>rs1129038<br>rs1800401<br>rs2238289<br>rs2240203<br>rs3935591<br>rs4778241<br>rs7183877<br>rs8028689<br>rs12593929<br>rs1800407<br>rs7495174

Table S1: Phenotypes considered for matching images to DNA, and corresponding SNPs .

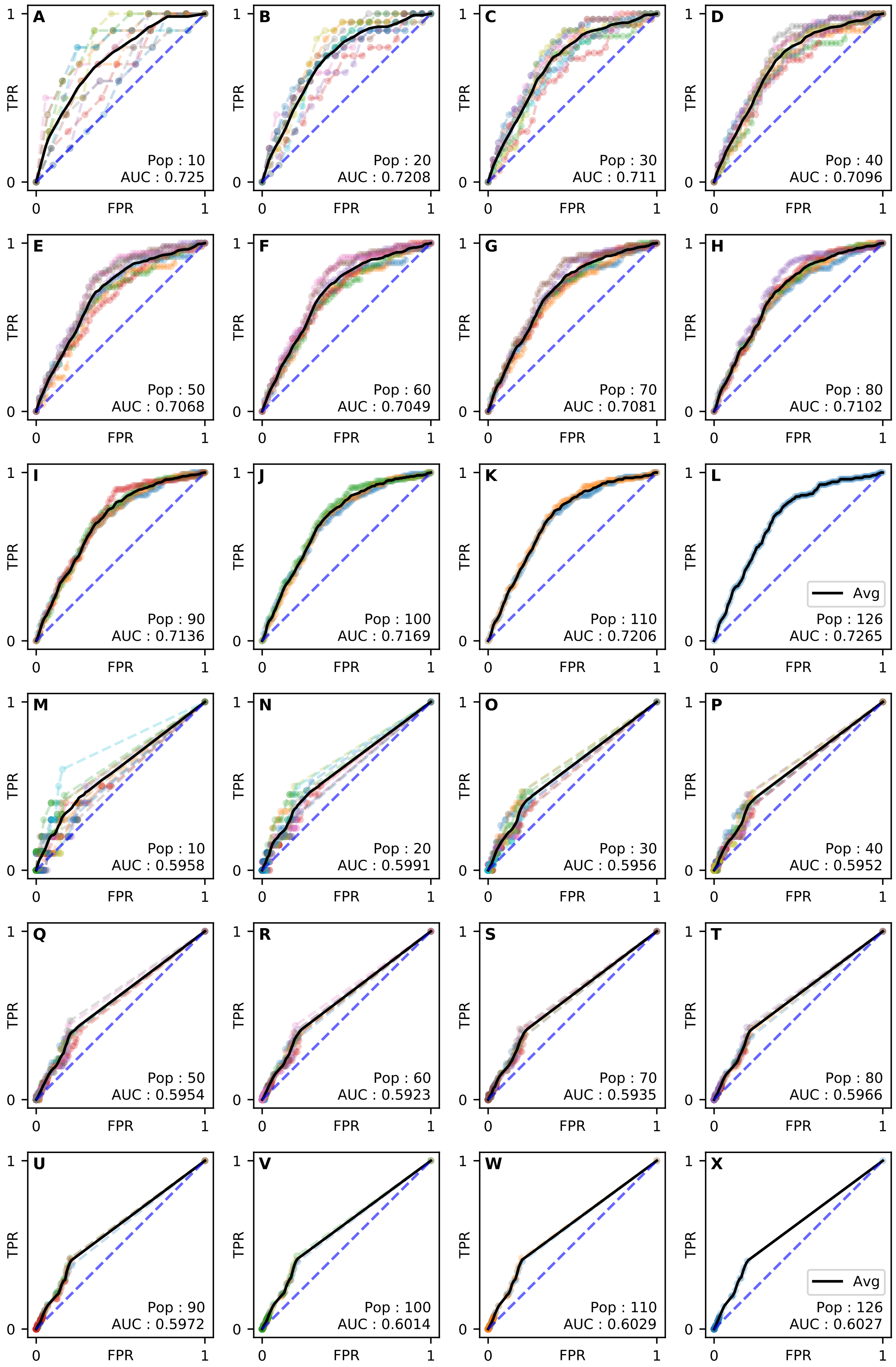

Fig. S1: **Receiver Operating Characteristic curves for various population sizes.** (A)-(L): Top k entries in the sorted list of DNA sequences per image are predicted to be matches. (M)-(X): Predictions are made independently for each image-DNA pair.

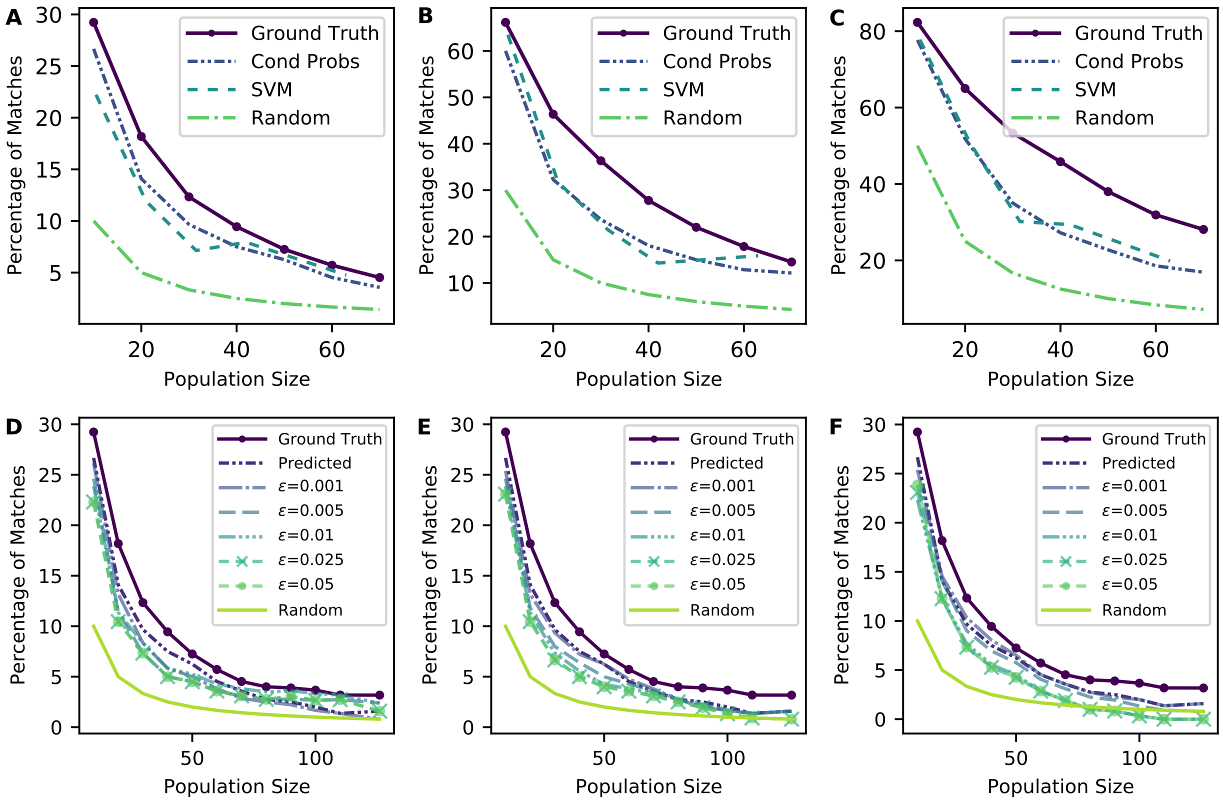

Fig. S2: **Using SVMs to fine-tune matching; Attacking phenotypes other than sex.** (A)-(C): Matching accuracy when SVMs are used on the 126 individuals to fine-tune. From left to right, (A) Top 1, (B) Top 3, (C) Top 5 matching accuracy with SVMs. The input to the SVM for an image-DNA pair is a vector of probabilities of phenotypes, where phenotypes are extracted from the image, and conditional probabilities are calculated from the DNA sequence, and the labels are binary indicating a match or otherwise. For each image in the test set, we rank all DNA sequences in order of their distance from the separating hyperplane, from most likely match to least likely match, and consider the top k as true matches. We note that this approach does not significantly improve matching accuracy, which we believe to be the result of low signal-noise ratio. (D)-(F): Top-1 matching performance when attacking phenotypes other than sex independently. While effective relative to clean images, attacking hair color, skin color or eye color alone does not reduce accuracy to below random except for fairly large populations, where matching accuracy is low to begin with.

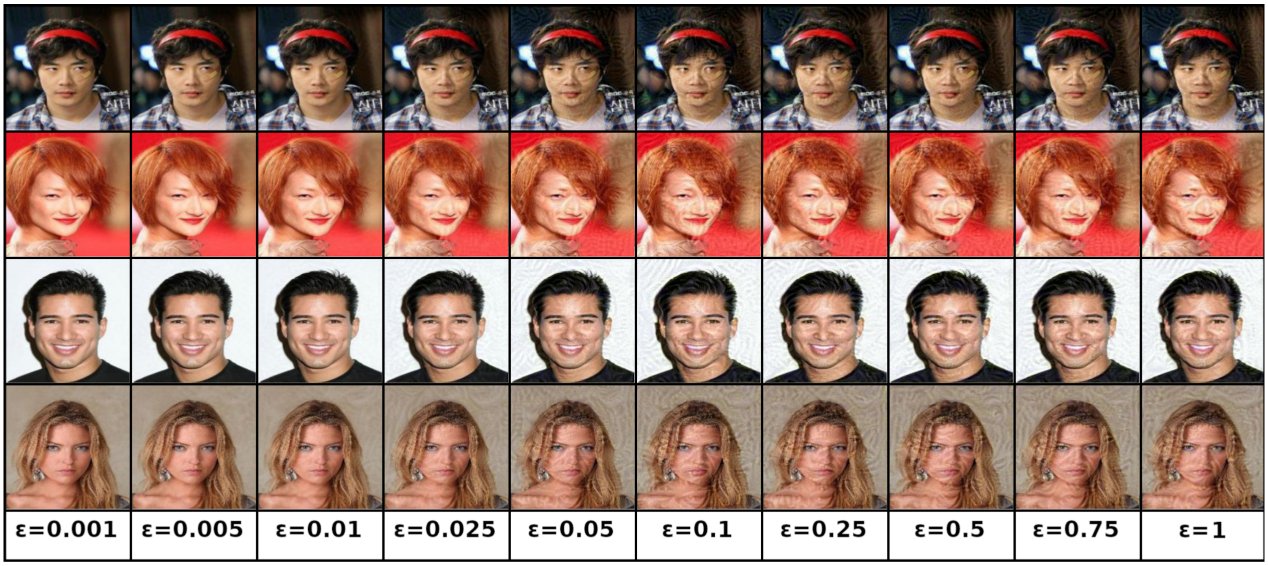

Fig. S3: **Impact of increasing maximum permitted perturbation, ϵ.** As the value of ϵ increases above 0.01, the noise added to the image becomes more visible.

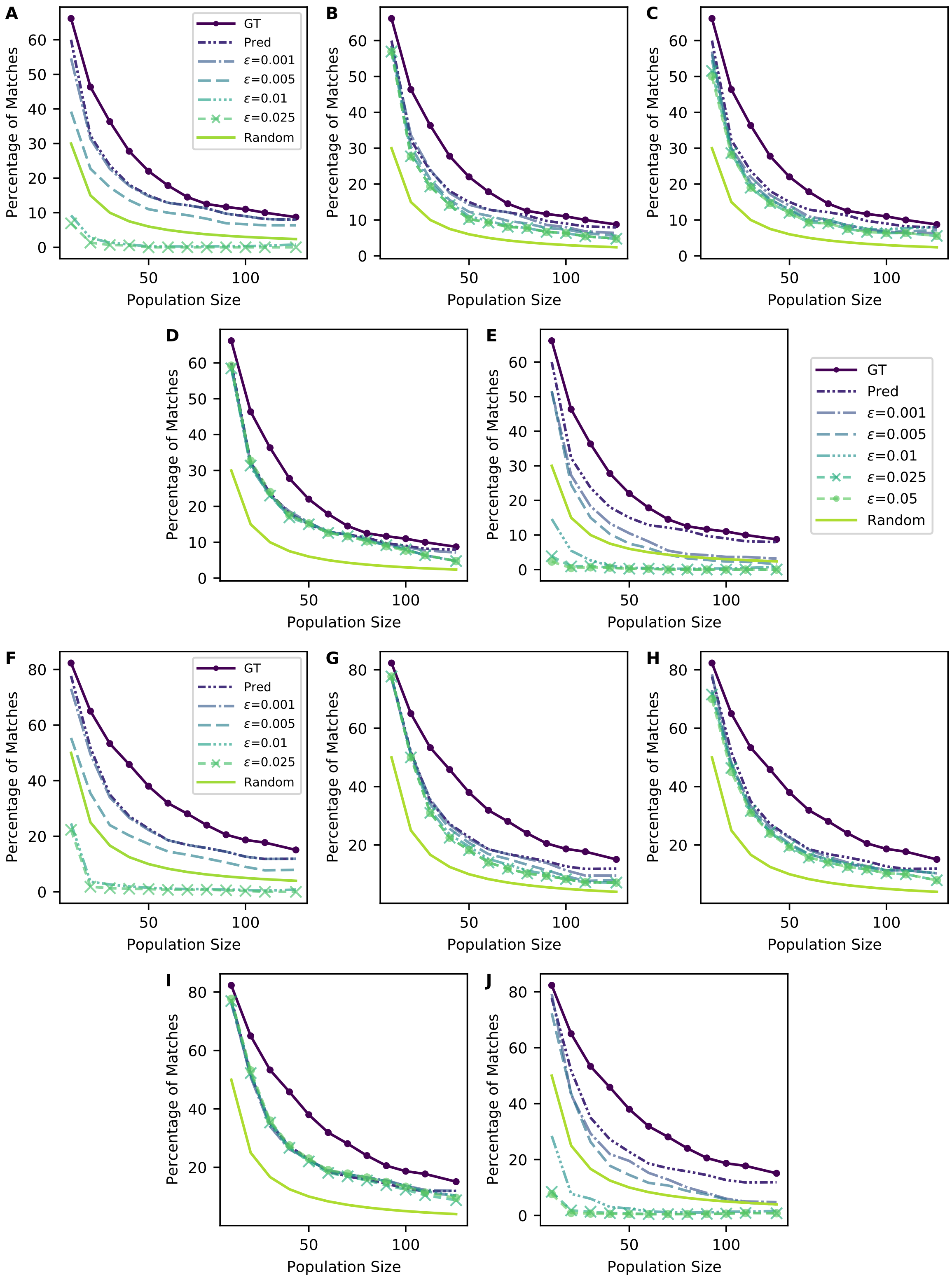

Fig. S4: **Matching accuracy with perturbed images.** (A)-(E): Accuracy of Top-3 matching with perturbed facial images, at different strengths of attack, i.e., values of ϵ, for (A) Sex, (B) Skin Color, (C) Eye Color, (D) Hair Color and (E) *Universal Noise*. Similar to the top-1 case, attacking sex is much more effective compared to attacking other phenotypes independently, and directly minimizing overall matching log-likelihood remains highly effective. (F)-(J): Accuracy of Top-5 matching with perturbed facial images, at different strengths of attack, i.e., values of ϵ, for (F) Sex, (G) Skin Color, (H) Eye Color, (I) Hair Color and (J) *Universal Noise*. Once again, while attacking sex manages to lower accuracy to below random for even small populations, attacking other phenotypes proves relatively ineffective. Yet again, direct minimization of the matching log-likelihood proves highly effective in preserving privacy.

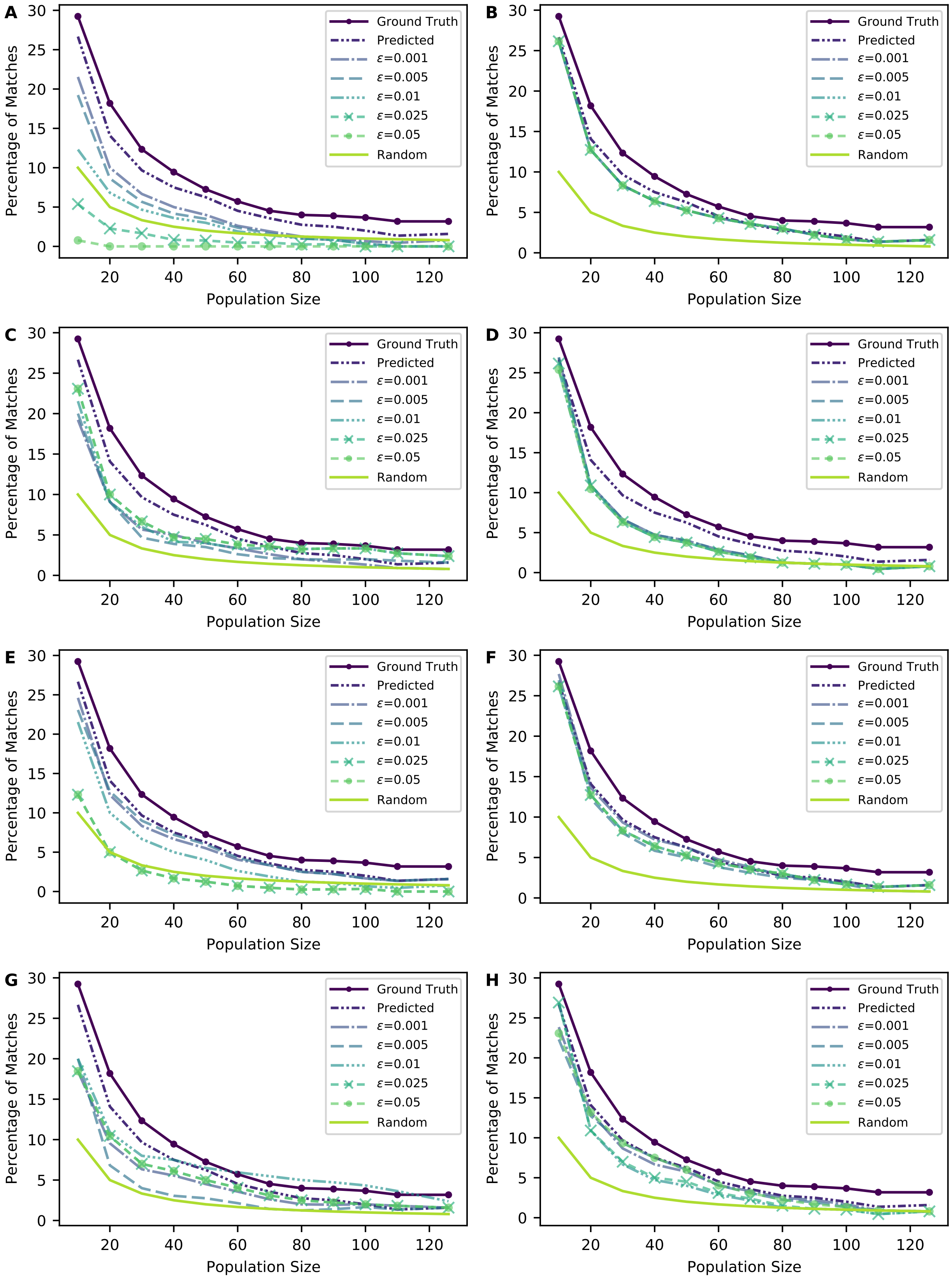

Fig. S5: **Impact of adversarial training.** (A)-(D): Top-1 Matching accuracy with robust classifiers, with images perturbed at ϵ between 0 and 0.05 for (A) Sex, (B) Skin Color, (C) Eye Color, (D) Hair Color. Each classifier was trained with adversarial examples at $\epsilon = 0.01$. (E)-(H): Top-1 Baseline accuracy of matching with adversarially trained classifiers, but clean images for (A) Sex, (B) Skin Color, (C) Eye Color, (D) Hair Color. Legends in each figure show the values of ϵ used for training the network.

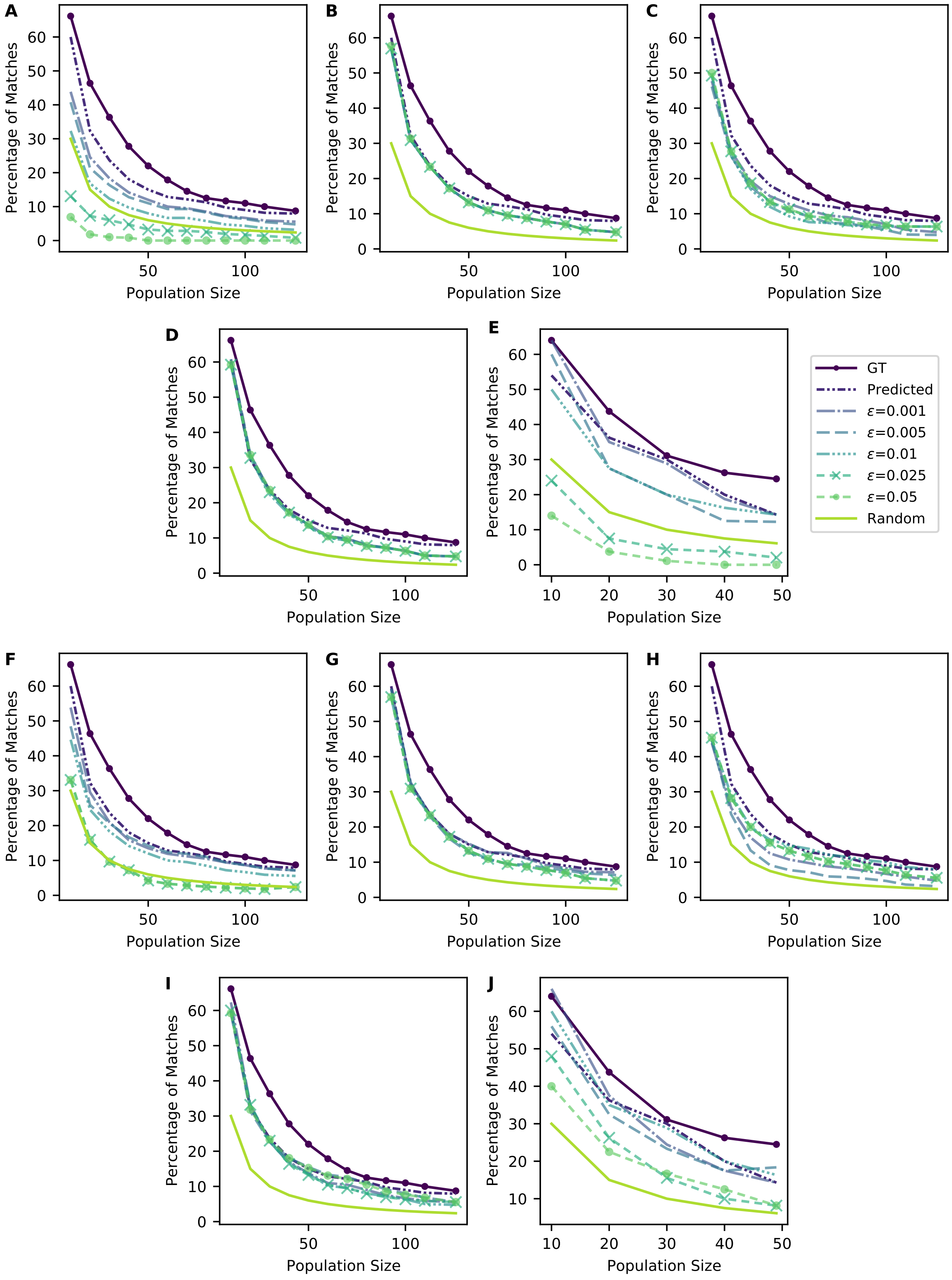

Fig. S6: **Impact of adversarial training on top-3 matching.**(A)-(E): Top-3 Matching accuracy with robust classifiers, with images perturbed at ϵ between 0 and 0.05 for (A) Sex, (B) Skin Color, (C) Eye Color, (D) Hair Color and (E) *Universal Noise*. Each classifier was trained with adversarial examples at $\epsilon = 0.01$. (F)-(J): Top-3 Baseline accuracy of matching with adversarially trained classifiers, but clean images for (A) Sex, (B) Skin Color, (C) Eye Color, (D) Hair Color and (E) *Universal Noise*. Legends in each figure show the values of ϵ used for training the network.

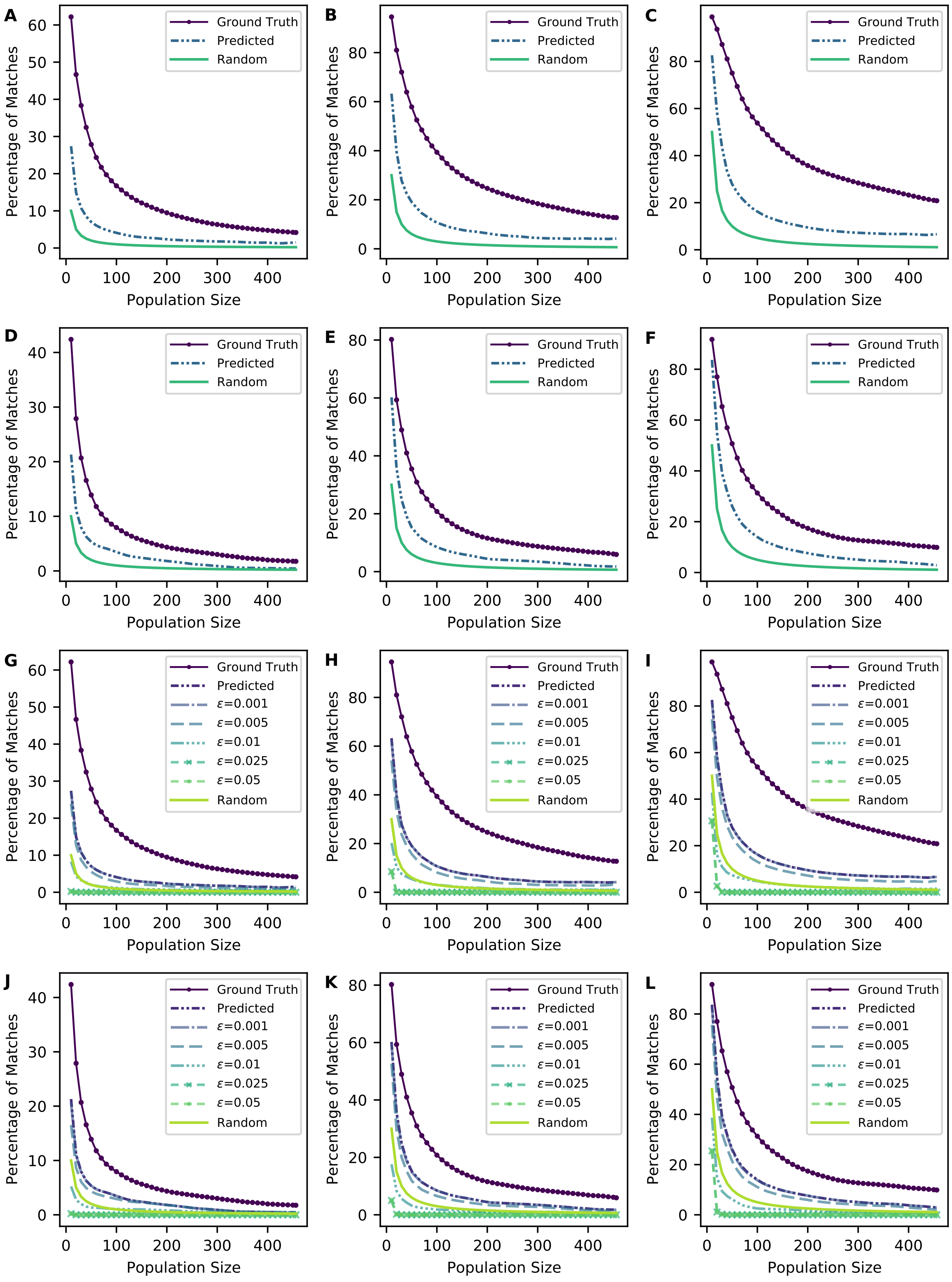

Fig. S7: **Matching accuracy with synthetic datasets and impact of attacking the sex-classifier.** (A)-(F): Matching Accuracy with the Ideal (top row) and Realistic (bottom row) synthetic datasets for top-1, top-3 and top-5. The ground truth accuracy is much higher in the ideal scenario - this is to be expected as genomes are explicitly picked to be the most representative of a corresponding image. The accuracy of predicted matches is much lower than results on the OpenSNP individuals would suggest. We narrow the cause of this down to the challenges in predicting eye-color from images. (G)-(L): Matching Accuracy with PGD-perturbed images targeting the sex-classifier, for the synthetic datasets for top-1, top-3 and top-5. Much like the OpenSNP data, fooling the prediction of sex from images proves to be a highly effective defense for the synthetic datasets, although at a higher value of $\epsilon = 0.025$.

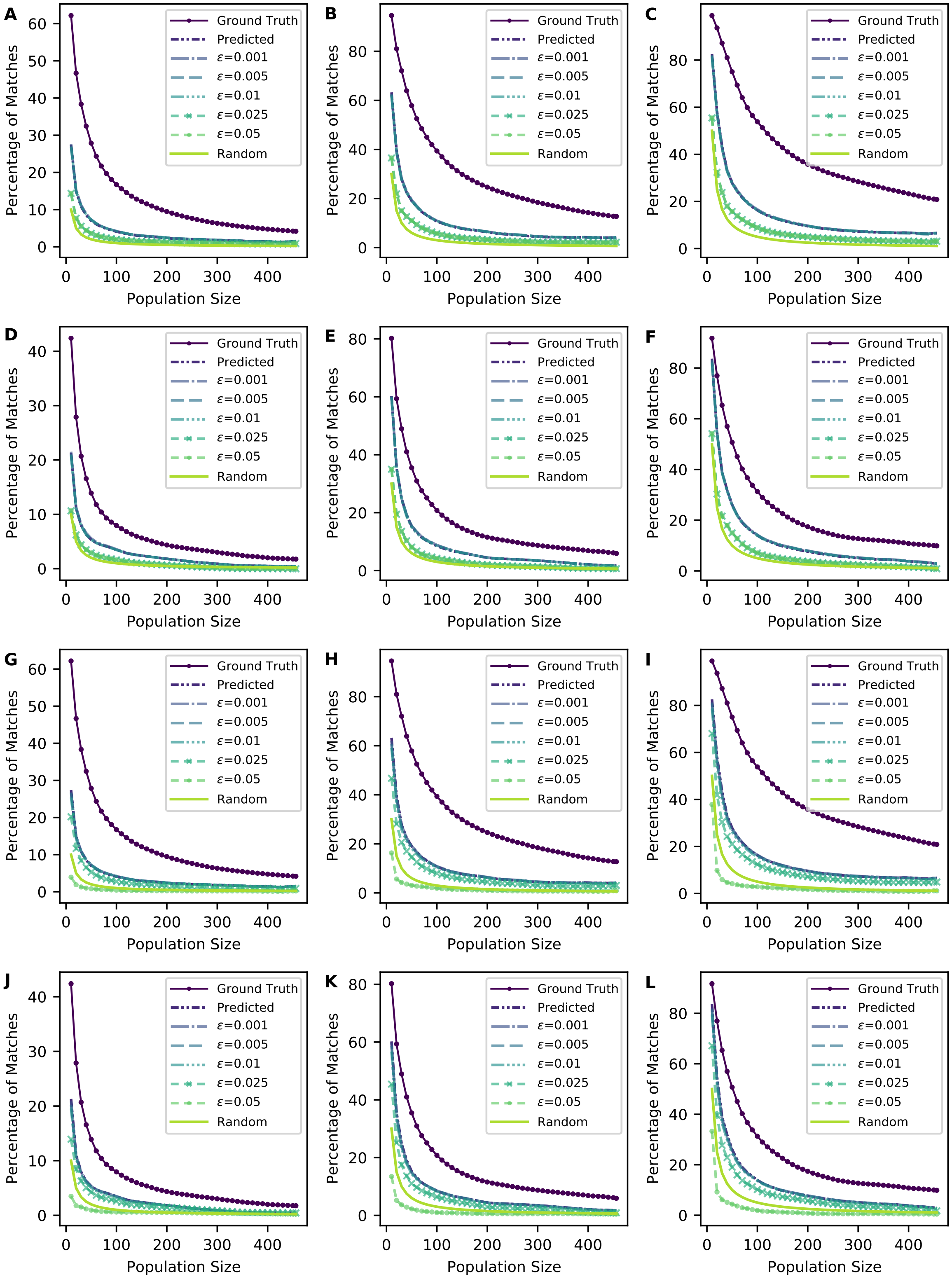

Fig. S8: **Impact of adversarially training the sex-classifier.** (A)-(F): Matching accuracy with sex-classifiers adversarially trained at various values of ϵ but using clean images for the ideal (top row) and and realistic (bottom row) synthetic datasets, for top-1, top-3 and top-5. Yet again, we observe that training against a strong enough adversarial noise attack incurs a significant performance penalty on clean images, making retraining detrimental to the malicious actor attempting re-identification. (G)-(L): Matching accuracy with a sex-classifier adversarially trained at $\epsilon = 0.01$, attacked with adversarial images for various values of ϵ, for the ideal (top row) and and realistic (bottom row) synthetic datasets, for top-1, top-3 and top-5. Similar to our results on the OpenSNP data, retraining boosts robustness to attacks of equal of lower strength as the retraining ϵ, but fail to be robust to stronger attacks. However, in contrast to the OpenSNP data, to reduce accuracy below random in this scenario requires an attack with $\epsilon = 0.05$, at which point adversarial noise starts to become visually evident.

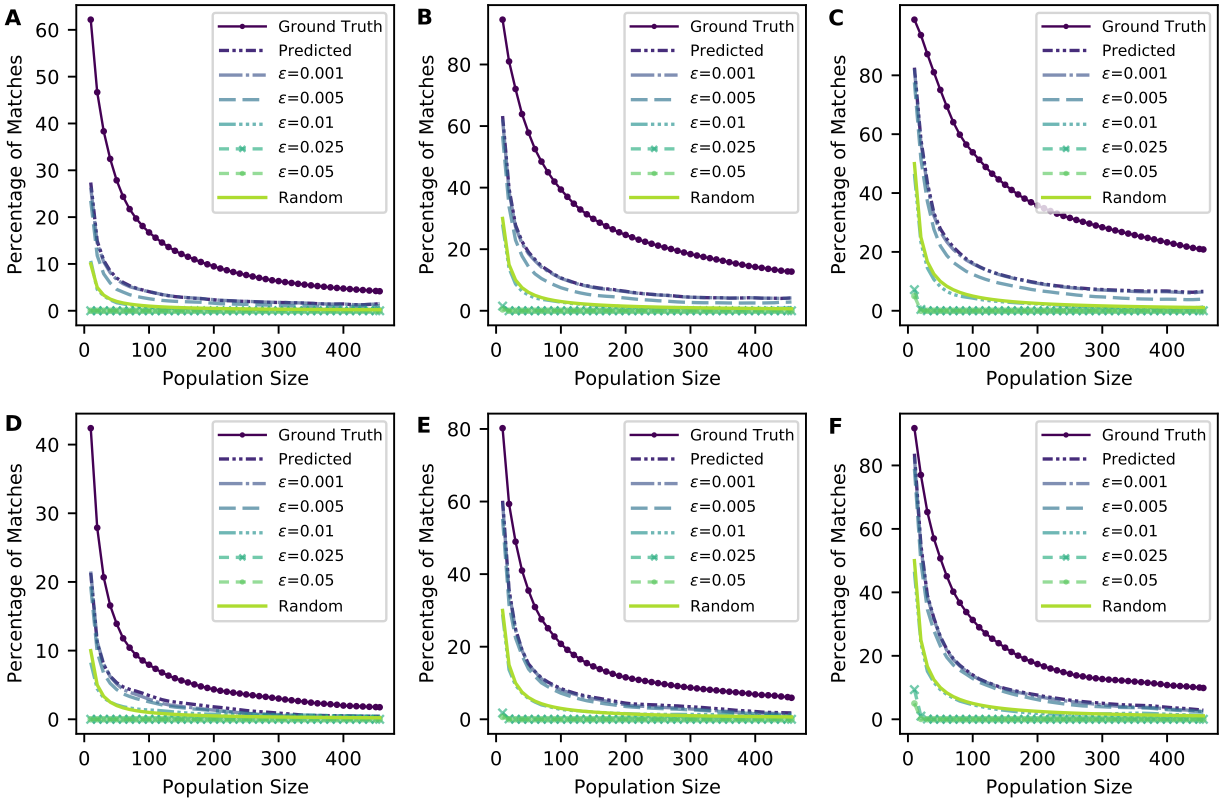

Fig. S9: **Protecting privacy by attacking all classifiers in parallel.** Matching Accuracy with images perturbed with *Universal Noise*, for the Ideal (top row) and Realistic (bottom row) synthetic datasets for top-1, top-3 and top-5. Here, the effectiveness of this method relative to simply attacking sex is much more pronounced. For a very reasonable attack ϵ of 0.01, the accuracy is reduced to near-random, and for attacks that are stronger, accuracy is reduced to zero, even for very small populations.

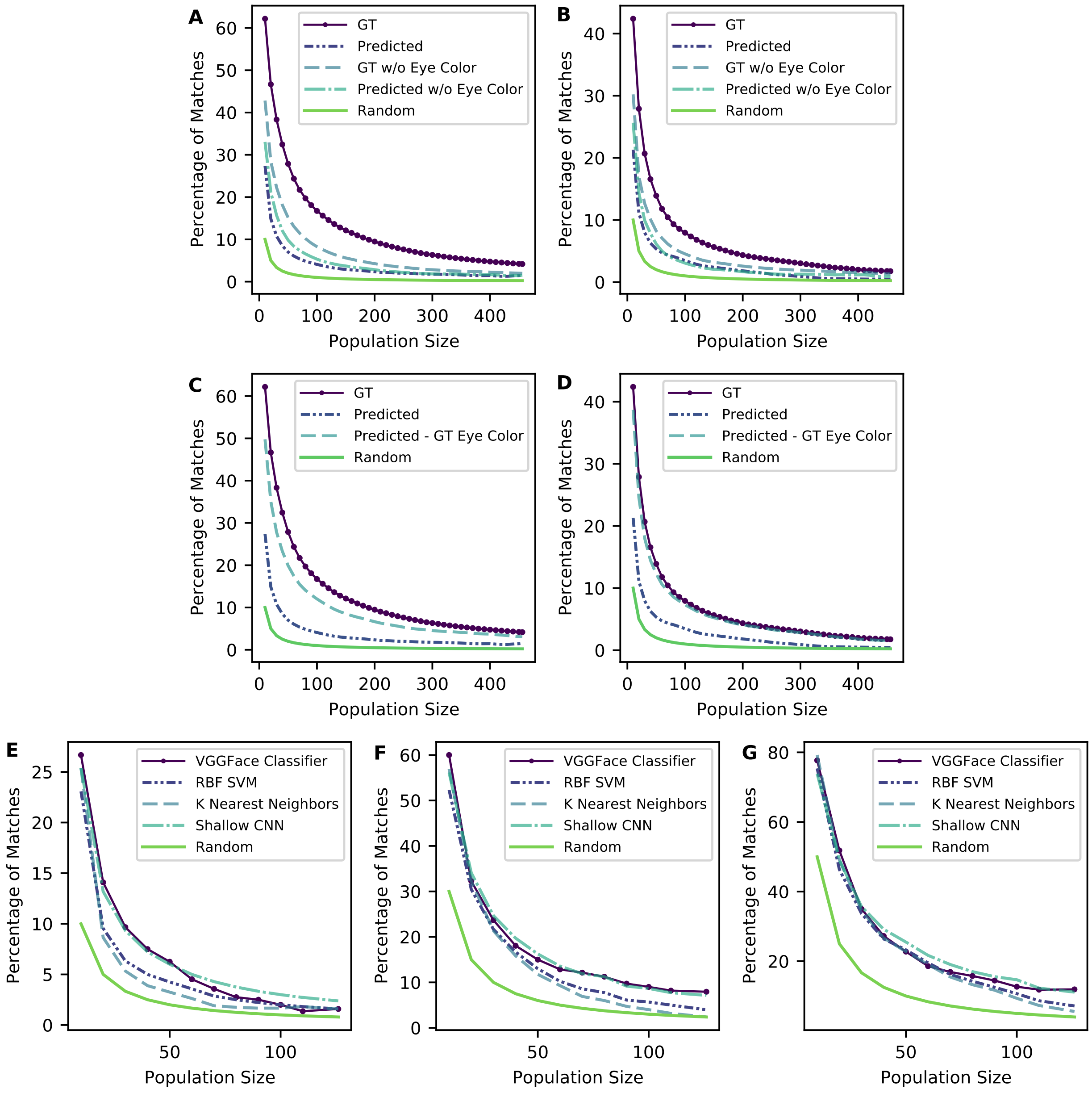

Fig. S10: **Effect of eye-color prediction accuracy.** (A)-(B): Matching accuracies with and without considering the eye-color phenotype, both predicted and ground-truth for (A) Ideal and (B) Realistic synthetic datasets. Notice the significant drop in ground-truth accuracy when eye-color is disregarded entirely. This points to the high importance of eye-color in matching. At the same time, notice that in the ideal dataset, disregarding eye-color entirely produces *better* matching accuracy than when including it, although it does not make a significant difference to the realistic dataset. This points to the presence of significant noise in our eye-color predictions. (C)-(D): Matching accuracies when the eye-color predictions from images are replaced by ground-truth values, for (C) Ideal and (D) Realistic synthetic datasets. The significant increase in matching accuracy (nearly the upper bound) in both synthetic datasets strongly suggests that we are limited in our matching ability by the poor performance of eye-color prediction. (E)-(G): Top-1, Top-3 and Top-5 matching accuracies with various eye-color prediction techniques. None of them are particularly effective.
